# A novel chemiluminescence sensor for sensitive detection of cholesterol based on the peroxidase-like activity of copper nanoclusters

**DOI:** 10.1038/srep39157

**Published:** 2016-12-14

**Authors:** Shuangjiao Xu, Yanqin Wang, Dayun Zhou, Meng Kuang, Dan Fang, Weihua Yang, Shoujun Wei, Lei Ma

**Affiliations:** 1 State Key Laboratory of Cotton Biology, Institute of Cotton Research of CAAS, Anyang 455000, China

## Abstract

A sensitive and selective chemiluminescence (CL) sensor based on the peroxidase-like activity of copper nanoclusters was established for the detection of cholesterol. Copper nanoclusters catalyse the CL reaction between luminol and H_2_O_2_. Because H_2_O_2_ is the oxidative product of cholesterol in the presence of cholesterol oxidase, the oxidation of cholesterol can be quantitatively converted to a CL response by combining the two reactions. The proposed method is simple and can be completed in a few minutes with high sensitivity. Under the optimal conditions, the CL intensity was proportional to the concentration of cholesterol over a wide range of 0.05–10 mM, with a detection limit of 1.5 μM. Furthermore, the method was successfully applied to determine cholesterol in milk powder and human serum with satisfactory accuracy and precision. This method expands the applications of nano-mimic enzymes in the field of CL-based sensors.

Cholesterol, a main lipid in humans, is an important structural component of the cell membrane, where it helps to maintain membrane fluidity and permeability. Because it also plays a vital role as a precursor in the production of Vitamin D and hormones, abnormal cholesterol levels cause certain diseases, such as anaemia, hypolipoproteinaemia, malnutrition hypertension, brain thrombosis, septicaemia and arteriosclerosis^1^,^2^,^3^. In humans, the sources of cholesterol are food and biosynthesis in the liver. Therefore, monitoring the cholesterol levels in food and blood is critical for disease control and prevention. Various analytical methods have been proposed to determine cholesterol content in many foods and biological samples, including liquid chromatograph^4^,^5^, spectrophotometric^6^,^7^,^8^, colorimetric^9^,^10^,^11^, electrogenerated chemiluminescence (ECL)^12^,^13^, and enzyme-linked immunosorbent assay^14^,^15^. The sensing platforms mentioned above has performed well for cholesterol detection, however, limitations still exists in the case of time consumption, low sensitivity and selectivity, and sophisticated instrumentation or standardization difficulties. Accordingly, it would be of great interest to develop cost-effective, ease-to-use and sensitive cholesterol sensor.

Chemiluminescence (CL) has received considerable attention by virtue of its simplicity, low detection limit, wide calibration range, and inexpensive instrumentation. Due to the combination of advantages of CL, Zhang *et al*. reported a sensor for free cholesterol based on immobilizing cholesterol oxidase onto sol-gel to generate an enzymatic reaction column^16^. Recently, CL studies have been extended to nanomaterial systems to enhance the inherent sensitivity and expand to novel applications of detection^17^,^18^,^19^. However, only a few nanomaterial-based CL methods are available for the determination of cholesterol. Chen *et al*. constructed a CL cholesterol sensor based upon the peroxidase-like activity of cupric oxide nanoparticles^20^. Also, Ehsani *et al*. relied on the catalytic activity of cupric oxide nanoparticles towards the luminol-H_2_O_2_ system using a Box-Behnken design for cholesterol determination^21^. However, these CL sensors are limited due to the complicated synthesis of the nanomaterials.

Copper nanoclusters (Cu NCs) consisting of several to tens of atoms have recently attracted much attention^22^,^23^. Xu *et al*. has demonstrated that Cu NCs could possess intrinsic peroxidase-like activity^24^. Compared to natural enzymes, Cu NCs show several advantages, such as ease of preparation, low cost, and high catalytic activity. Additionally, we have found that Cu NCs could greatly enhance the CL of the luminol-H_2_O_2_ system in strongly alkaline media^25^. Moreover, hydrogen peroxide is a product of the cholesterol oxidase-catalysed reaction of cholesterol and oxygen. According to the three aforementioned points, we have established a novel, simple, and sensitive sensor for the detection of cholesterol by combining the highly selective enzymatic reaction with the sensitive chemiluminescence system catalysed by copper nanoclusters in this work.

## Experimental

### Reagents and materials

A 1.0 × 10^−2 ^mol L^−1^ stock solution of luminol (3-aminophthalhydrazide) was prepared by dissolving luminol (Sigma) in a 0.1 mol L^−1^ sodium hydroxide solution and stored at 4 °C. Working solutions of luminol were prepared by diluting the stock solution with ultra-pure water. Bovine serum albumin (BSA) was purchased from Sigma Sangon Biotech Co., Ltd. (Shanghai, China). CuSO_4_**·**5H_2_O, sodium hydroxide, isopropanol, Triton X-100, cholesterol and ChOx were purchased from Sigma-Aldrich Co., Ltd. (USA). A stock solution of cholesterol was prepared by dissolving cholesterol in a mixture of isopropanol and Triton X-100 (1:1, v/v), and the standard cholesterol solutions were diluted with PBS (pH 7.4) and then stored at 4 °C. All of the reagents were used as purchased without purification, and ultra-pure water was used throughout.

### Synthesis of BSA–Cu nanoclusters

BSA modified Cu NCs were prepared in aqueous solution following the previous reported method^26^. In a typical experiment, 1 mL aqueous CuSO_4_**·**5H_2_O solution (20 mM) was added to BSA solution (5 mL, 15 mg mL^−1^) under vigorous stirring for 5 min at room temperature. Then, the solution pH was adjusted to 12 by adding NaOH solution and the mixture was allowed to proceed under vigorous stirring at 55 °C for 8 h. The solution was then dialyzed in ultra-pure water for 48 h to remove unreacted Cu^2+^. The final solution was stored at 4 °C in refrigerator when not in use.

### General procedure for CL analysis

The chemiluminescence detection was conducted on a laboratory-built flow injection CL system, consisting of a model IFFM-E flow injection system (Xi’an Remex Co.), a model IFFA-S multifunctional CL detector (Xi’an Remex Co.), and a computer, as shown in Figure S1. Two peristaltic pumps with three channels were used to carry the reactants to the flow cell. One peristaltic pump was used to carry Cu NCs and sample solutions with two channels, and the other pump was used to deliver luminol solution at 1.9 mL/min. The CL signals produced were monitored by a photomultiplier tube and then the output signals were obtained by a computer automatically. Data acquisition and treatment were performed with Remex software running under Windows XP. When the CL system was used to investigate the effects of interference compounds, one peristaltic pump was used to deliver Cu NCs and the mixture of sample and luminol with two channels, and the other pump was used to carry the interference compound solution at 1.9 mL/min.

### Quantitative analysis of cholesterol

The cholesterol catalytic reaction was carried out by adding 65 μL of cholesterol (0.05 mM to 10 mM), 25 μL of 30 U ml^−1^ cholesterol oxidase (ChO*x*), and 162.5 μL of 0.1 mol/L phosphate buffer solution (pH 7.4) into an EP tube. The mixture was then incubated at 37 °C for 10 min to obtain the testing sample solution. Before CL testing, the sample solutions were diluted 10 times by water. The assay was applied to milk and human serum samples for the determination of cholesterol.

### Preparation of samples

#### Milk powder sample

2.0 g of milk powder was dissolved in 10 mL of KOH/ethanol solution and saponified in a water bath for 1 h. Then, 10 mL water and 20 mL *n*-hexane were added into the sample solution, and the mixture was centrifuged at 5000 rpm for 5 min. Finally the *n*-hexane was separated, and the solvent was evaporated under a stream of nitrogen. The residue was redissolved with the previous mixture of isopropanol and Triton X-100.

#### Human serum sample

For determination of free cholesterol, 0.2 mL of a human serum sample was diluted with 1.8 mL ethanol solution and 2.0 mL water. Each of these 20-fold diluted samples were mixed with 4.0 mL *n*-hexane and then centrifuged for 5 min. Finally, the *n*-hexane extract was separated, and the solvent evaporated under a stream of nitrogen. The residue was redissolved with the previous mixture of isopropanol and Triton X-100.

## Results and Discussion

### Principle for the chemiluminescence detection of cholesterol

The UV-vis and fluorescence spectra of as-prepared Cu NCs are shown in Figures S2 and S3. When the Cu NCs were excited at 325 nm, they showed an emission peak centred at 410 nm. The features of the obtained spectra were in keeping with previous reports, indicating the successful preparation of Cu NCs.

**Figure 1:**
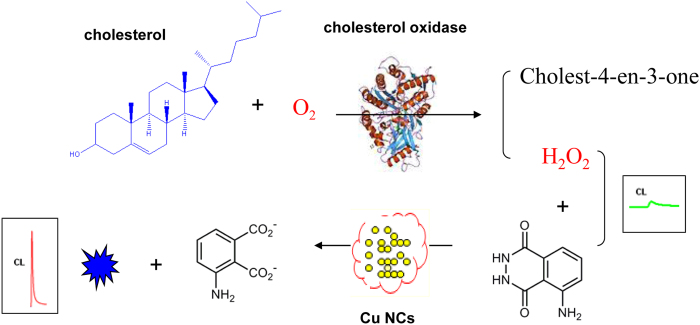
Principle of the Cu NCs-based chemiluminescence sensor for cholesterol.

The strategy of this chemiluminescent cholesterol sensor has been suggested to occur in three steps, as shown in Fig. 1. Firstly, H_2_O_2_ is generated from the oxidation of cholesterol in the presence of cholesterol oxidase. Secondly, the chemical reaction of luminol with H_2_O_2_ is accelerated by the catalytic effect of Cu NCs, resulting in the formation of excited-state 3-aminophthalate anions which emit light (λ_max _= 425 nm) on relaxation to the ground state (see Equations 1 and 2). Finally, the relationship between the concentration of cholesterol and the intensity of the CL signal was evaluated for the sensor.


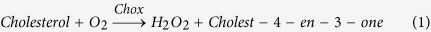



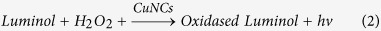


### Chemiluminescence analysis under different experimental conditions

In our recent work, we studied the possible mechanism of the luminol-H_2_O_2_-Cu NC systems using the CL spectra, UV-visible spectroscopy and radical scavengers^25^. Initially, The O-O bond of H_2_O_2_ might be broken up into two OH• radicals via the catalysis of Cu NCs. Next, the OH• radicals are thought to react with the luminol anion and HO_2_^−^ to form the luminol radical and superoxide radical anion O_2_^•−^, which further react with each other to form the excited 3-aminophthalate anion (3-APA*). As a result, the CL signal is enhanced significantly. In this work, H_2_O_2_ originated from the oxidation of cholesterol by O_2_ in the presence of ChO*x*.

**Figure 2:**
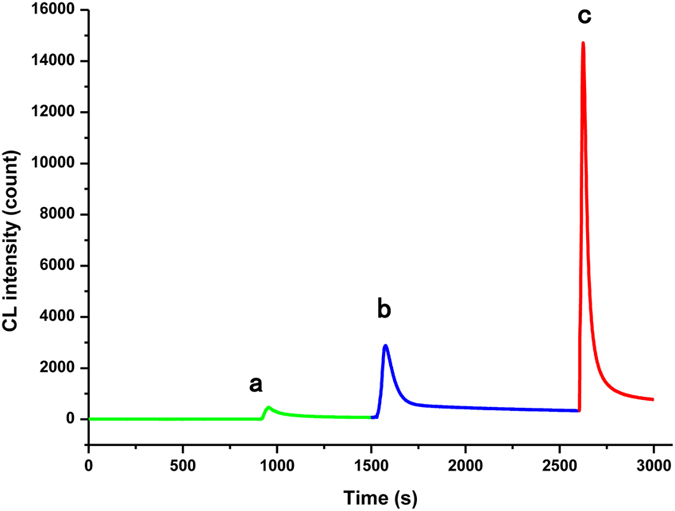
Kinetic curves of luminol CL system under different conditions. (**a**) luminol + cholesterol/ChO*x*; (**b**) luminol + cholesterol/ChO*x* + CuSO_4_; (**c**) luminol + cholesterol/ChO*x* + Cu NCs. Luminol solution: 6 × 10^−4^ mol L^−1^ (pH 12), Cu NCs : 6.4 mg L^−1^, ChO*x* :30 U mL^−1^. cholesterol :10 mM, Cu^2+^: 20 mM.

In order to prove the consistency of mechanism, the kinetic curves of CL systems for different situations were considered. A batch method was used to study the reaction of luminol, cholesterol, ChO*x* and Cu NCs. The addition of Cu NCs to luminol and cholesterol (or ChO*x*) did not produce light. When cholesterol and ChO*x* was simultaneously injected into the luminol system without Cu NCs, the CL intensity increased slightly due to the formation of H_2_O_2_ (Fig. 2a). Importantly, a remarkable CL enhancement (up to 31-fold in 20 s) was found when Cu NCs were introduced into the luminol-cholesterol/ChO*x* system, and the light lasted for approximately 200 s (Fig. 2c). Compared with Cu NCs, the CL intensity was only enhanced by approximately 5-fold by a CuSO_4_ solution (Fig. 2b). These results indicate that the significantly enhanced CL in Fig. 1c is related directly to Cu NCs. The CL spectra of luminol-cholesterol/ChO*x* in the presence of Cu NCs is shown in Figure S4. The maximum emission wavelength of luminol-cholesterol/ChO*x*-Cu NCs was found to be about 425 nm, suggesting that the luminophor for the CL system is still the excited 3-aminophthalate anion. Therefore, the addition of cholesterol/ChO*x* into the luminol system containing Cu NCs does not generate a new luminophor, which is consistent with previous reports.

### Optimization of the experimental conditions

**Figure 3:**
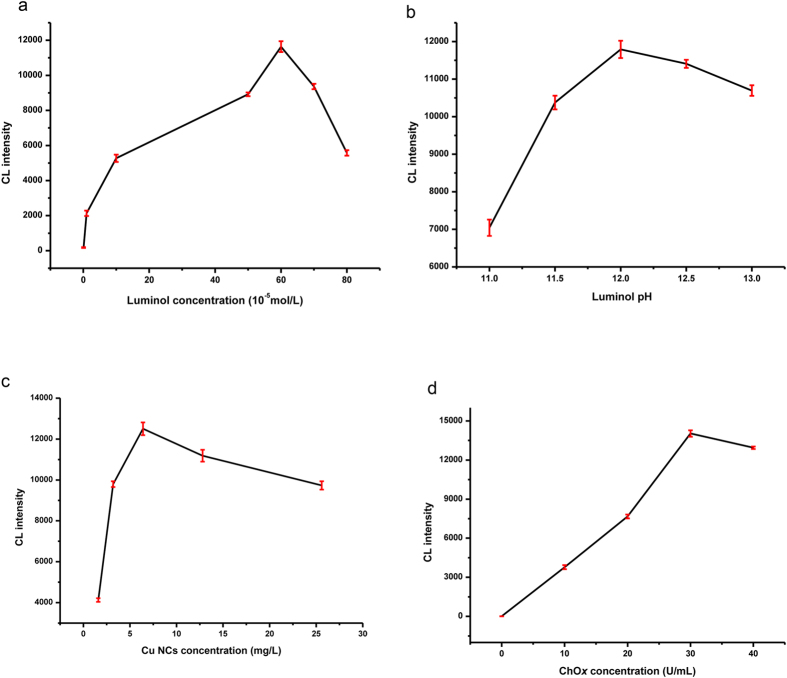
Effects of different concentration of reagents and pH on the CL intensity.

The cholesterol sensor was investigated under varying concentrations of luminol, ChO*x*, and Cu NCs as well as luminol solutions of differing pH. As shown in Fig. 3, the CL intensity increased with increasing luminol concentration from 1 × 10^−6^ to 6 × 10^−4^ mol L^−1^ (Fig. 3a), but a higher concentration of luminol led to self-absorption of the emitted radiation and a decrease in the CL intensity. Because the pH of the luminol solution is a key factor in the generation of CL, the effect of solution pH values from 11 to 13 on the sensor performance was studied (Fig. 3b). At pH values lower than 12, the CL intensity increased with increasing pH, but the opposite trends was observed at pH values higher than 12. The effect of the Cu NC concentration was also tested (Fig. 3c), and the optimized concentration was found to be 6.4 mg L^−1^ Cu NCs. ChO*x* plays an important role in forming H_2_O_2_, which leads to CL emission. Therefore, the impact of the ChO*x* concentration was studied over the range of 0–40 U ml^−1^, and it was found that the CL intensity increased with increasing concentration of enzyme (Fig. 3d). However, considering the CL intensity and reagent consumption, the optimized conditions for the CL system were as follows: 6 × 10^−4^ mol L^−1^ luminol in NaOH solution (pH 12) with 6.4 mg L^−1^ Cu NCs and 30 U mL^−1^ ChO*x*.

### Cholesterol detection based on chemiluminescence sensor

**Figure 4:**
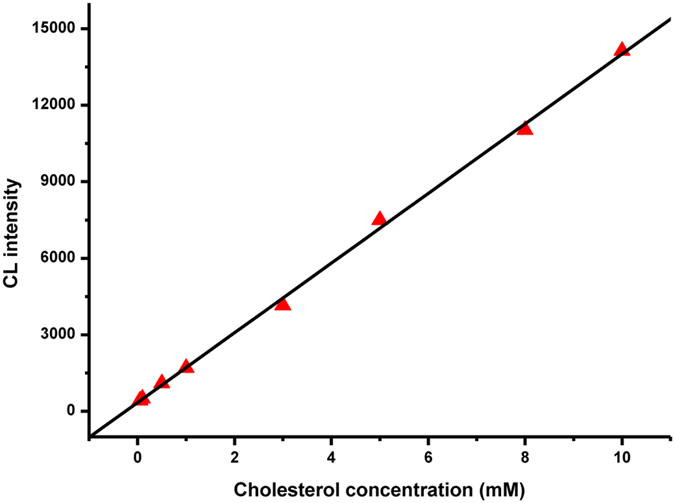
The linear calibration plot of the CL reaction for cholesterol detection.

The CL sensor was evaluated under the optimal experimental conditions described above by detecting standard cholesterol solutions. As shown in Fig. 4, the CL intensity was found to increase with increasing cholesterol concentrations. The linear calibration range encompassed over 3 orders of magnitude from 0.05 mM to 10 mM with a relatively low detection limit of 1.5 μM (LOD, S/N = 3), and the regression equation was *I* = 1366.2*c* + 343.8 with a correlation coefficient R = 0.9993 (n = 8) (where *c* is the cholesterol concentration in mM). The relative standard deviation (RSD) was 2.4% for 1 mM cholesterol (n = 11). Furthermore, the performance of the proposed CL sensor was compared with previously reported cholesterol assays in terms of LOD and linear range (Table 1). It could be observed that the proposed CL sensor showed a high sensitivity for the determination of cholesterol.

### Selectivity and stability of the chemiluminescence sensor

**Figure 5:**
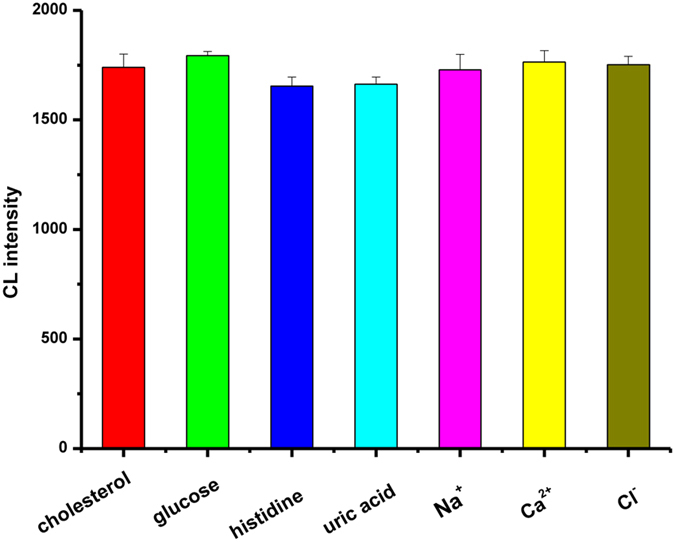
The interference effect of 1 mM glucose, 0.1 mM histidine, 0.11 mM uric acid, 10 mM Na^+^, 10 mM Ca^2+^ and 10 mM Cl^−^ in the detection of 1 mM cholesterol in 0.1 mol/L phosphate buffer solution (pH 7.4).

Selectivity and stability are important parameters for examining the performance of a sensor. To study the selectivity of the method towards the detection of cholesterol, a standard solution of 1 mM cholesterol was subjected to varying amounts of possible interferents. The tolerable limit was considered as a relative error less than the 5% level. As shown in Fig. 5, interferents, such as the organic molecules glucose, histidine, and uric acid and the inorganic ions Na^+^, Ca^2+^, and Cl^−^, had a negligible effect on the detection of 1 mM cholesterol, demonstrating that ChO*x* has a selectivity for cholesterol catalysis. The stability of the sensor was also studied by measuring the change in the CL signal at regular intervals of 2 days for 2 weeks. The CL signal decreased to 90% after 1 week and 75% after 2 weeks. These results show that the sensor has good selectivity and stability.

### Application of the chemiluminescence sensor

To explore potential applications of the method, the content of cholesterol in commercial milk and human serum samples was determined. The milk and human serum samples were first pre-treated according to previous reports^20^. Next, recovery experiments were carried out to evaluate the practical applicability of the method by adding a known amount of cholesterol to the real samples. As shown in Table 2, the recoveries of the spiked milk samples and human serum samples ranged from 91.0% to 103.9% with the RSD less than 5.0%, indicating that the CL sensor is reliable for the detection of cholesterol in real samples.

## Conclusion

In summary, we have constructed a novel chemiluminescent cholesterol sensor based on the peroxidase-like activity of copper nanoclusters. The sensor showed good performance for cholesterol detection with the advantages of high sensitivity, selectivity, stability, an acceptable linear range of 0.05–10 mM and a relatively low detection limit (1.5 μM). Moreover, the method was successfully applied to the determination of cholesterol in milk and human serum samples. This work is also expected to widen the application of enzyme-catalysed chemiluminescence reactions in bioanalysis.

## Additional Information

**How to cite this article**: Xu, S. *et al*. A novel chemiluminescence sensor for sensitive detection of cholesterol based on the peroxidase-like activity of copper nanoclusters. *Sci. Rep.* **6**, 39157; doi: 10.1038/srep39157 (2016).

**Publisher’s note:** Springer Nature remains neutral with regard to jurisdictional claims in published maps and institutional affiliations.

## Supplementary Material

Supplementary Information

## Figures and Tables

**Table 1:** Comparison of the analytical performance of proposed method with others.

Method	Material	Linear range (mM)	Detecton limit (mM)
Colorimetry^11^	ZnO NPs-CNTs	0.5–500^a^	0.2^a^
Chronoamper-ometry^27^	ChOx-CS/Hb-CS	0.01–0.6	0.0095
Amperometry^28^	Pt-ZnO Nanospheres	2.78–12.2	0.5
CV^29^	(PDDA-[MWCNTs-ChO*x*]_5_)	0.02–1	0.03
ECL1^30^	Ag NPs	1–700	0.65
ECL2^31^	Au NPs	0.033–1	0.0011
ECL3^32^	CdSeTe/ZnS QD	0.25–5	Not given
CL1^21^	CuO NPs	0.025–7.17	0.0064
CL2	Cu NCs	0.05–10	0.0015

^a^nmol/L.

**Table 2:** Evaluation of cholesterol determination in milk and human serum samples by standard addition method.

Samples	Added (mM )	Found (mM )	Recovery (%)	RSD (%)
Milk sample 1	0	0.071	—	2.5
	0.1	0.162	91.0	2.4
	0.5	0.540	93.8	3.0
Milk sample 2	0	0.062	—	2.0
	0.1	0.156	94.0	3.5
	0.5	0.646	98.0	2.8
Serum sample 1	0	1.850	—	4.3
	5	6.981	102.3	4.5
	10	11.963	101.1	3.2
Serum sample 2	0	1.197	—	3.7
	5	6.390	103.9	4.2
	10	11.120	99.2	4.0

(n = 3).
