# Supplementary material for: A novel chemiluminescence sensor for sensitive detection of cholesterol based on the peroxidase-like activity of copper nanoclusters
Source: Sci Rep. 2016 Dec 14;6:39157. doi: 10.1038/srep39157 (PMC5155213; doi:10.1038/srep39157)
Supplement: Supplementary Information [file srep39157-s1.pdf]

A novel chemiluminescence sensor for sensitive detection of cholesterol based on the peroxidase-like activity of copper nanoclusters

Shuangjiao Xu[†], Yanqin Wang[†], Dayun Zhou, Meng Kuang, Dan Fang, Weihua Yang, Shoujun Wei*, Lei Ma*

State Key Laboratory of Cotton Biology, Institute of Cotton Research of CAAS, Anyang 455000, China

† These authors contributed equally to this work.

*__Corresponding Author__

Lei Ma, Ph.D, Email:malei2002@163.com, Phone: 86-372-2562278,

Fax numbers: 86-372-2562278

*__Co-corresponding Author:__

Shoujun Wei, Email: 13503728390@163.com

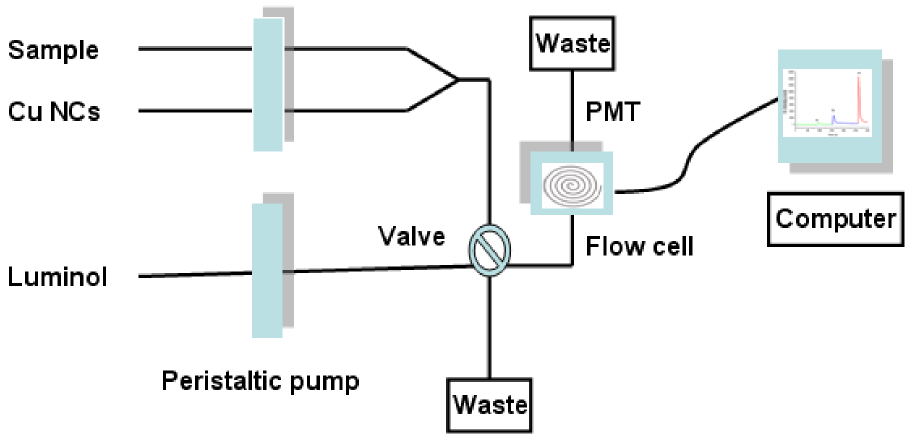

Figure S1. Schematic diagram of the flow injection CL sensing system.

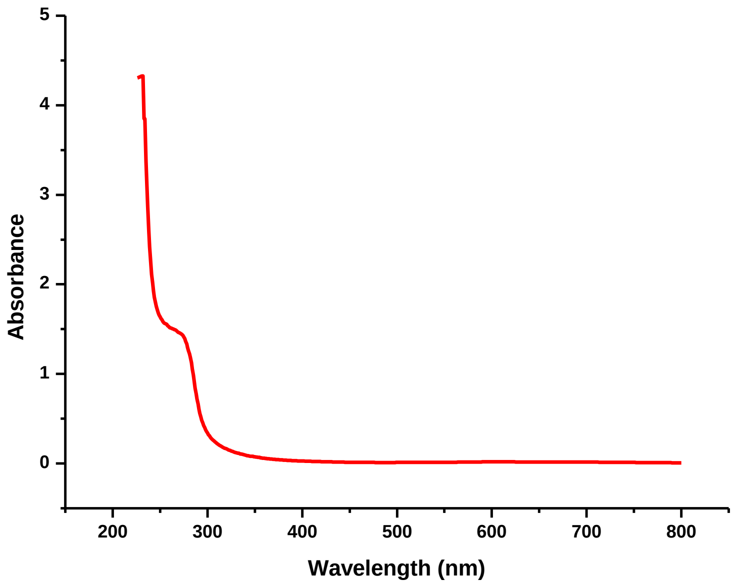

Figure S2. The UV-vis spectra of as-prepared Cu NCs.

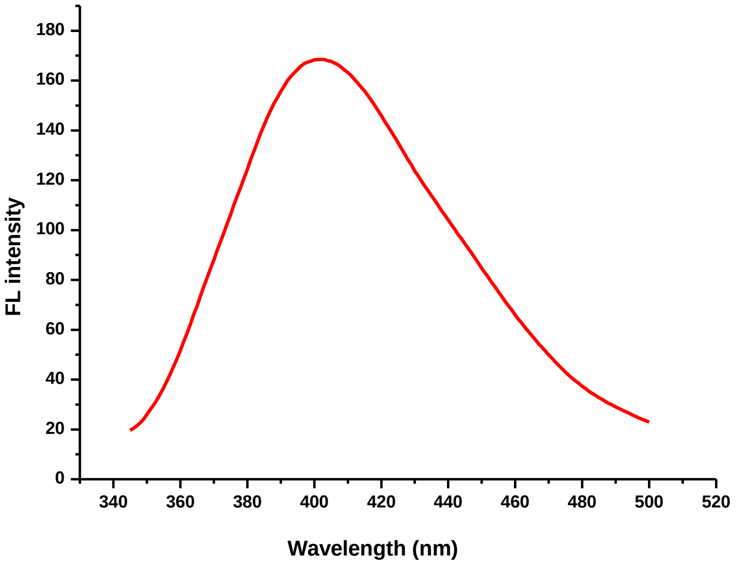

Figure S3. The fluorescence emission spectra of as-prepared Cu NCs (Ex=325nm).

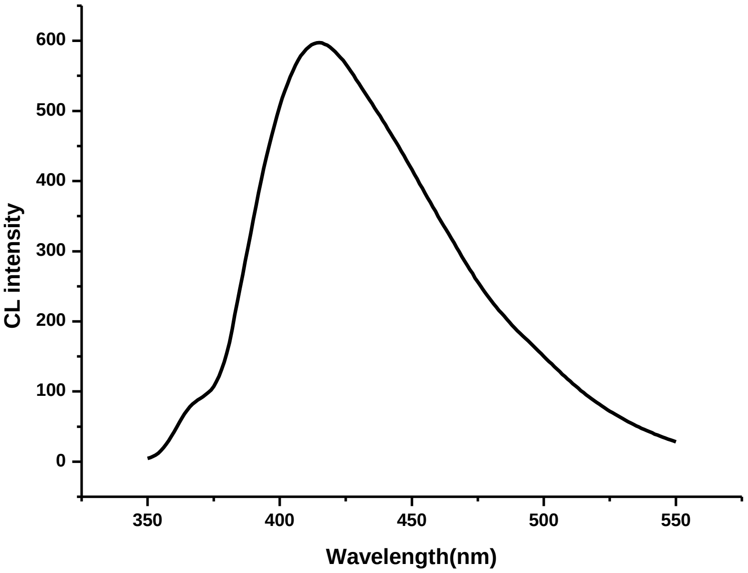

Figure S4. Chemiluminescence spectrum for the luminol –cholesterol/ChO*x*-Cu NCs CL system.
